# PD-1 expression in hepatocellular carcinoma predicts liver-directed therapy response and bridge-to-transplant survival

**DOI:** 10.1007/s00262-021-03087-z

**Published:** 2021-10-24

**Authors:** Kelley G. Núñez, Tyler Sandow, Daniel Fort, Mina Hibino, Paige Wright, Ari J. Cohen, Paul T. Thevenot

**Affiliations:** 1 Institute of Translational Research, Ochsner Clinic Foundation, New Orleans, LA 1520 Jefferson Highway USA; 2 Interventional Radiology, Ochsner Clinic Foundation, New Orleans, LA 1514 Jefferson Highway USA; 3 Centers for Outcomes and Health Services Research, Ochsner Clinic Foundation, New Orleans, LA 1514 Jefferson Highway USA; 4 Multi-Organ Transplant Institute, Ochsner Clinic Foundation, New Orleans, LA 1514 Jefferson Highway USA; 5 Faculty of Medicine, University of Queensland, Brisbane, Australia

**Keywords:** Hepatocellular carcinoma, PD-1, Immunotherapy, Liver-directed therapy

## Abstract

**Background:**

Hepatocellular carcinoma (HCC) patients undergo liver-directed therapy (LDT) to control tumor burden while awaiting liver transplantation with response impacting waitlist survival. In this study, we investigate the link between absolute lymphocyte count (ALC) and PD-1 expression with response to LDT and bridge-to-transplant survival.

**Methods:**

Treatment-naïve HCC patients (*n* = 86) undergoing LDT were enrolled at a single center from August 2016–March 2020. Response to LDT was determined using mRECIST. Blood samples were collected on the day of LDT and at follow-up. Cells were analyzed for phenotype by flow cytometry. Outcomes were liver transplantation or tumor progression.

**Results:**

Incomplete response to initial LDT was associated with tumor progression precluding liver transplantation (OR: 7.6, 1.7 – 33.3, *P* < 0.001). Univariate analysis of baseline *T* cell phenotypes revealed ALC (OR: 0.44, 0.24–0.85, *P* = 0.009) as well as intermediate expression of PD-1 on CD4 (OR: 3.3, 1.03–10.3, *P* = 0.034) and CD8 *T* cells (OR: 3.0, 0.99–8.8 *P* = 0.043) associated with incomplete response to LDT. Elevations in PD-1 expression were associated with increased risk of bridge-to-transplant tumor progression (HR: 3.2, 1.2–9.4). In patients successfully bridged to liver transplantation, pre-treatment peripheral PD-1 profile was associated with advanced tumor staging (*P* < 0.005) with 2/4 of patients with elevations in PD-1 having T3-T4 TNM staging compared to 0 with low PD-1 expression.

**Conclusion:**

Low lymphocyte count or elevated expression of the PD-1 checkpoint inhibitor is associated with incomplete response to LDT and increased risk of bridge-to-transplant tumor progression. Patients with impaired *T* cell homeostasis may benefit from PD-1 immunotherapy to improve response to LDT and improve bridge-to-transplant outcomes.

**Supplementary Information:**

The online version contains supplementary material available at 10.1007/s00262-021-03087-z.

## Introduction

Hepatocellular carcinoma (HCC) is the fourth leading cause of cancer-related deaths worldwide. In the Barcelona Clinic Liver Cancer staging system, treatment options include partial liver resection, liver transplantation, liver-directed therapies, and systemic therapies dependent upon tumor location and severity of liver disease. Orthotopic liver transplantation (OLT) is the preferred treatment option for eligible candidates as both the underlying cirrhosis and cancer are treated. While early detection of HCC has increased referrals to liver transplantation and improved overall survival in HCC, patients are often required to undergo liver-directed therapy (LDT) while awaiting OLT to reduce tumor burden within transplant criteria. LDT has proven to be an effective strategy for both downstaging and bridge-to-transplant by increasing waitlist survival and outcomes as well as reducing post-transplant HCC recurrence [1–3]. Response to LDT has been used as a surrogate to evaluate tumor biological aggressiveness with studies linking LDT response to tumor size and grade impacting post-LT recurrence risk [2, 4–6], bridge-to-transplant survival [6], post-transplant outcome [7], and overall survival after OLT [8, 9]. Patients non-responsive to LDT have higher tumor grades at explant compared to those with partial or complete responses [4]. Those patients that achieve complete radiographic response are at lowest risk of dropout [6, 10], have higher rates of complete pathological necrosis, and recurrence-free survival [11, 12]. While complete radiographic response decreases the risk of viable tumor at the time of transplant, two recent retrospective studies found the rates of complete pathologic necrosis was found in < 25% of patients with pre-transplant complete radiographic responses [6, 11]. However, the treatment itself may potentially cause tumor recurrence or promote resistance. LDT may also select for aggressive clones within the tumor with altered growth and neovascularization patterns that radiographically mimic a treated lesion. This is evidenced by the discord between pre-transplant imaging and pathology findings in the explant liver. Primary tumor burden and the HCC biomarker, a-fetoprotein (AFP), have been the only factors linked to response to LDT. Their combined sensitivity for predicting tumor response is low and complicated by challenges in controlling for treatment modality and mixed cohorts of early stage with intermediate/late stage HCC [13, 14].

Low pre-treatment absolute lymphocyte counts were shown to be associated with poor response to initial LDT [4] indicating the immune system’s involvement in treatment response. The factors that ultimately drive initial response to LDT are not well understood. The complication of cirrhosis-associated immune dysfunction persists in HCC patients affecting both local and systemic immune cells. Cirrhosis-induced immunodeficiency could impact anti-tumoral response and waitlist outcomes. Investigations of *T* cell health in HCC patients undergoing LDT remain limited and would give insight into treatment-resistance. *T* cells expressing the exhausted marker programmed cell death protein 1 (PD-1) might prevent appropriate activation for anti-tumoral response. A study showed patients with elevated PD-1 expression on tumor-infiltrating CD8 *T* cells were correlated with poor prognosis [15]. In this study, we investigate baseline factors in treatment-naïve HCC patients awaiting liver transplantation associated with initial response to LDT and their impact on bridge-to-transplant survival.

## Methods

### Patient cohort

**Fig. 1:**
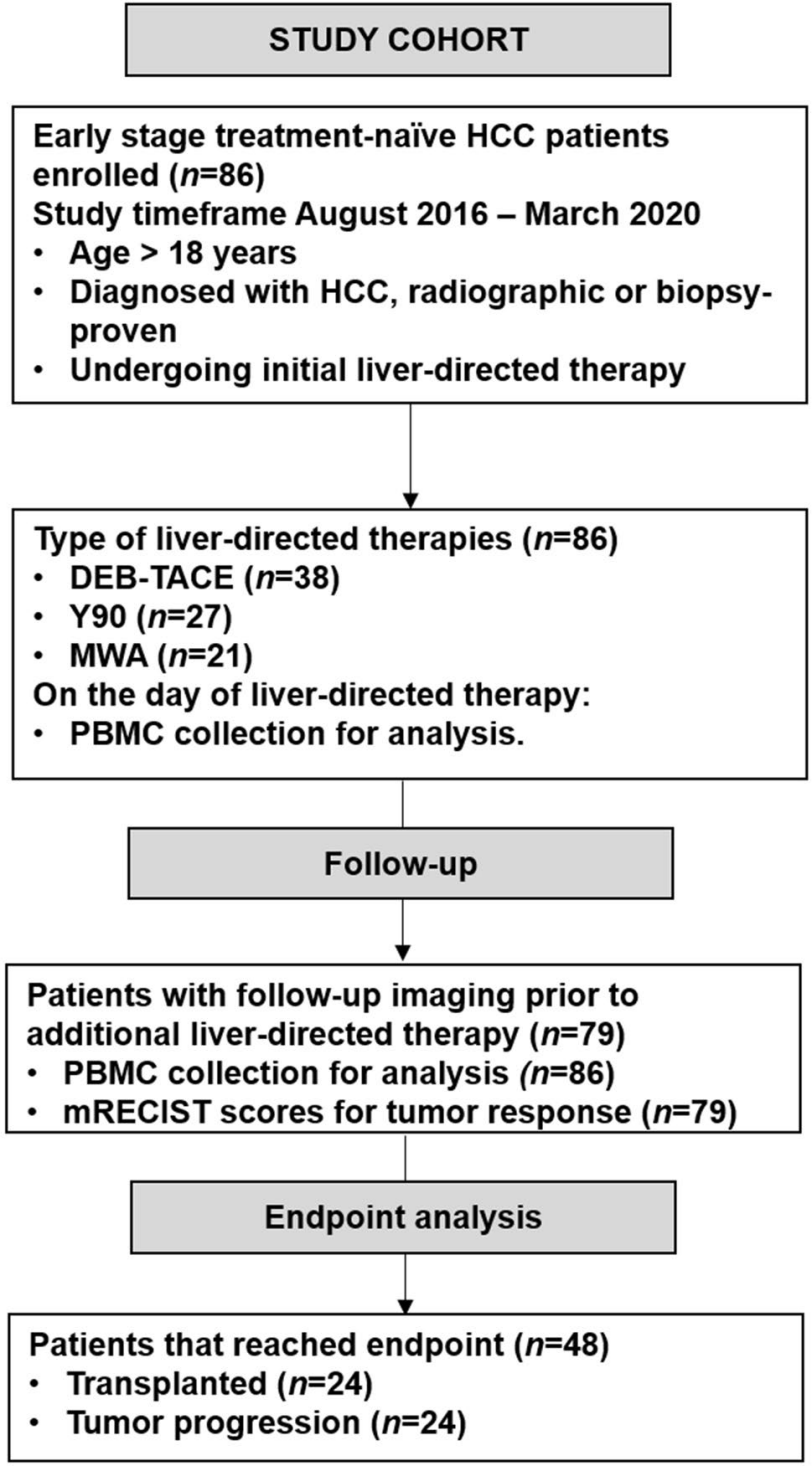
CONSORT-style flow diagram. Study design of treatment-naïve HCC patients undergoing initial liver-directed therapy as a bridge-to-transplantation. DEB-TACE, doxorubicin-eluting beads transarterial chemoembolization; Y90, Yttrium-90; MWA, microwave ablation; PBMC, peripheral blood mononucleated cells; mRECIST, modified response evaluation criteria in solid tumors

A prospective, single-center study was performed after institutional review board approval was conducted in accordance with ethical guidelines set forth by the 1975 Declaration of Helsinki. Written informed consent was obtained prior to blood collection and liver-directed therapy. Cohort included eighty-six treatment-naïve patients diagnosed with HCC and undergoing liver-directed therapy between August 2016 and March 2020. Enrollment criteria and study outline are shown in Fig. 1. Initial HCC diagnosis was determined radiographically or through biopsy according to American Association for the Study of Liver Disease guidelines [16] and analyzed by board-certified radiologist and pathologist, respectively. Variables extracted from the electronic medical record included: demographics [age, sex, and race], liver cirrhosis etiology, complete metabolic panel [sodium, creatinine, albumin, and bilirubin], international ratio (INR), complete blood counts [white blood count, polymorphonucleated count, absolute lymphocyte count, and platelets], alpha-fetoprotein (AFP), Model for End-Stage Liver Disease-Sodium (MELD-Na) score, viral hepatitis status [active, cure/remission, or non-viral], and radiographic measurements of tumor burden [lesion size and number] at the time of initial LDT. History of decompensation of cirrhosis was defined as having one or more of the following conditions: ascites, jaundice, hepatic encephalopathy, and/or esophageal varices with bleeding. Cohort was then grouped based on having one or more decompensating events (present) or none (absent). The presence of portal hypertension and splenomegaly was determined via computed tomography or magnetic resonance imaging.

### Liver-directed therapy and evaluation of tumor response

Choice of liver-directed therapy modality was made during a multidisciplinary liver conference comprised of interventional radiologists, hepatologists, and liver transplant surgeons. Three LDT modalities are offered at institution were doxorubicin-eluting bead transarterial chemoembolization (DEB-TACE), microwave ablation (MWA), and Yttrium-90 (Y90). For inclusion in the study, patients presented at a multidisciplinary liver conference were deemed bridge-to-transplant candidates. The bridge-to-transplant study population was Barcelona Clinic Liver Cancer Stage A-B (*n* = 82) or had T1-T2 HCC burden and met institutional criteria for downstaging (*n* = 4). Downstaging criteria also included: total bilirubin < 4 mg/dL, serum creatinine < 1.5 mg/dL, without main portal vein thrombus or extrahepatic malignancy, and absent of gross ascites based on ultrasound or computed tomography. Selection of LDT was based on performance status and tumor characteristics (size and location).

Routine follow-up imaging was obtained either using triple-phase computed tomography or magnetic resonance imaging with contrast. Response to LDT was determined using modified response criteria for solid tumors (mRECIST) criteria [17] by board-certified interventional radiologist with > 10 years’ experience. Briefly, mRECIST is broken down into four categories based on arterial enhancement of the targeted lesion: complete (CR), partial (PR), stable disease (SD), or disease progression (DP). Complete response (CR) was defined as the disappearance of arterial enhancement within the targeted lesion in the absence of new lesion. Incomplete response was defined as PR, SD, or DP. Response to mRECIST was grouped as either initial or durable. Initial mRECIST classification was based on < 3-month follow-up imaging for DEB-TACE and MWA with < 6-months for Y90. Durable mRECIST response is based on routine imaging 90–180 days post-LDT provided no additional LDT was performed. Patients that underwent surveillance imaging 3–6 months after initial treatment without a second LDT intervention in between had their mRECIST score reevaluated to determine a durable response. Durable mRECIST response was used if available, if not, initial response was used. Durable responses were chosen over initial to allow for LDT to fully take effect. Based on response to initial LDT, patients would either undergo surveillance with additional imaging after 3 months or receive additional therapy.

### Blood collection, PBMC isolation, and flow cytometry

On the day of LDT, peripheral blood was collected immediately prior to procedure and again at routine imagining follow-up. Peripheral blood mononucleated cells (PBMCs) were isolated by Ficoll gradient then cryopreserved. PBMCs were thawed and used directly for staining for flow cytometry. Cells were stained with the following surface antibodies to determine *T* cell lineage: anti-CD3 FITC (clone UCHT1), anti-CD56 PE (clone CMSSB), anti-CD8 PerCp Cy5.5 (clone RPA-T8), anti-CD57 PE Cy7 (clone NK-1), anti-PD-1 APC (clone J105), anti-CD45 AF700 (clone 2D1), and anti-CD4 APC-eFluor 780 (clone RPA-T4). Surface markers were detected using Attune NxT (ThermoFisher) and analyzed using Attune NxT Software (ThermoFisher). Absolute CD4 and CD8 counts were determined by multiplying the absolute lymphocyte count (ALC) by the percentage of CD4/CD8 obtained via flow cytometry.

### Outcomes

Primary study outcomes were bridge-to-transplant survival based on either orthotopic liver transplantation or dropout due to tumor progression. Tumor progression as monitored after initial LDT and defined as progression beyond Milan Criteria (defined as 1 lesion > 5 cm or up to 3 lesions all ≥ 3 cm, extrahepatic spread, or tumor thrombus), or AFP exceeding 1000 ng/mL. Censored events included: lost to follow-up, dropout due to non-tumor causes, patient choice to decline OLT, or non-tumor related death on waitlist. Among patients who underwent orthotopic liver transplantation, liver explant was evaluated for pathological staging based on TMN staging according to AJCC 8th edition along with the presence of lymphovascular invasion and satellite nodules by board-certified pathologists.

### Statistics

Continuous variables are listed as median with interquartile range (IQR) while categorical variables are listed as percentage of the total. Chi-square and Fisher’s exact test were used to analyze categorical variables. Bridge-to-transplant survival was calculated using the log-ranked Kaplan–Meier method. Logistic regression analysis was used for factors associated with bridge-to-transplant outcomes and initial response to liver-directed therapy. Univariate factors were deemed significant for *P* value < 0.100 and evaluated in multivariate analysis. Milan criteria instead of largest lesion size were used for multivariate analysis. Multivariate analysis of factors associated with bridge-to-transplant survival was performed using Cox proportional hazards model with significance determined by likelihood ratio test. Matched pairs analysis with nonparametric Wilcoxon signed-rank test was used to determine significant differences in factors at baseline and post-treatment. One-way ANOVA with Tukey multiple comparison test was used to determine significance between ALC at different times during waitlist period. Analyses were performed using SAS JMP version 13.0 (SAS Institute, Cary, NC) or GraphPad Prism version 8.2.0 (San Diego, CA).

## Results

### Treatment naïve HCC patient cohort

Eligible patients (*n* = 86) undergoing an initial LDT as bridge-to-liver transplantation were enrolled from August 2016 to March 2020 (Fig. 1). Cohort demographics, baseline clinical labs, and radiographic measures of tumor burden are listed in Supplemental Table 1. Cohort was 65% Caucasian with median age of 63 years and predominantly male (70%). Primary liver cirrhosis etiology was hepatitis C virus (HCV, 51%) followed by HCV and alcoholic steatohepatitis (HCV + EtOH, 20%), and nonalcoholic steatohepatitis (NASH, 13%). Tumor burden was 81% within Milan criteria established from lesion size. The HCC biomarker, AFP, had a median of 15 ng/mL with 34% of cohort over the established threshold of 50 ng/mL prior to LDT. First-line LDT included doxorubicin-eluting beads transarterial chemoembolization (DEB-TACE) in 44% of cohort, followed by Yttrium-90 radioembolization (Y90, 31%), and microwave thermal ablation (MWA, 25%).

**Table 1:** Univariate Analysis of Demographics and Tumor Burden with Bridge-to-Transplant Outcomes

	Bridge-to-transplant outcome					
	Univariate			Multivariate		
Demographic	*P* value	OR	95% CI	*P* value	OR	95% CI
Cirrhosis etiology	0.490					
HCV						
HCV-EtOH						
NASH						
EtOH						
Other						
Sex, male vs. female	0.504					
Age, years	0.555					
Tumor burden						
Multifocal, yes vs. no	0.504					
Largest Lesion size, cm	** < 0.001**	3.5	1.6**–**7.9			
Cumulative lesion size, cm	0.144					
Milan, outside vs. inside	**0.028**	5.5	1.0**–**29.5	0.082	6.7	0.8**–**56.9
Hepatocellular carcinoma biomarker						
AFP, ≥ 50 ng/mL	**0.012**	5.2	1.3**–**20.1	**0.018**	9.2	1.5**–**58.0
Laboratory values						
Sodium, mmol/L	0.793					
Creatinine, mg/dL 1.0	0.154					
Albumin, g/dL	0.474					
Bilirubin, mg/dL	0.423					
INR	0.104					
MELD-Na, at time of LDT	0.182					
Liver-directed therapy						
Initial LDT type	0.461					
DEB-TACE						
MWA						
Y90						
Response to initial LDT, incomplete vs. complete	**0.003**	7.6	1.7**–**33.3	**0.033**	7.4	1.2**–**46.3

OR, Odds ratio; CI, confidence interval; response to initial LDT as incomplete includes mRECIST responses of partial response, stable disease, and progress of disease. Milan criteria was included in multivariate analysis in lieu of largest lesion size

Bold value indicates *P* < 0.05

### Response to initial liver-directed therapy and bridge-to-transplant survival

**Fig. 2:**
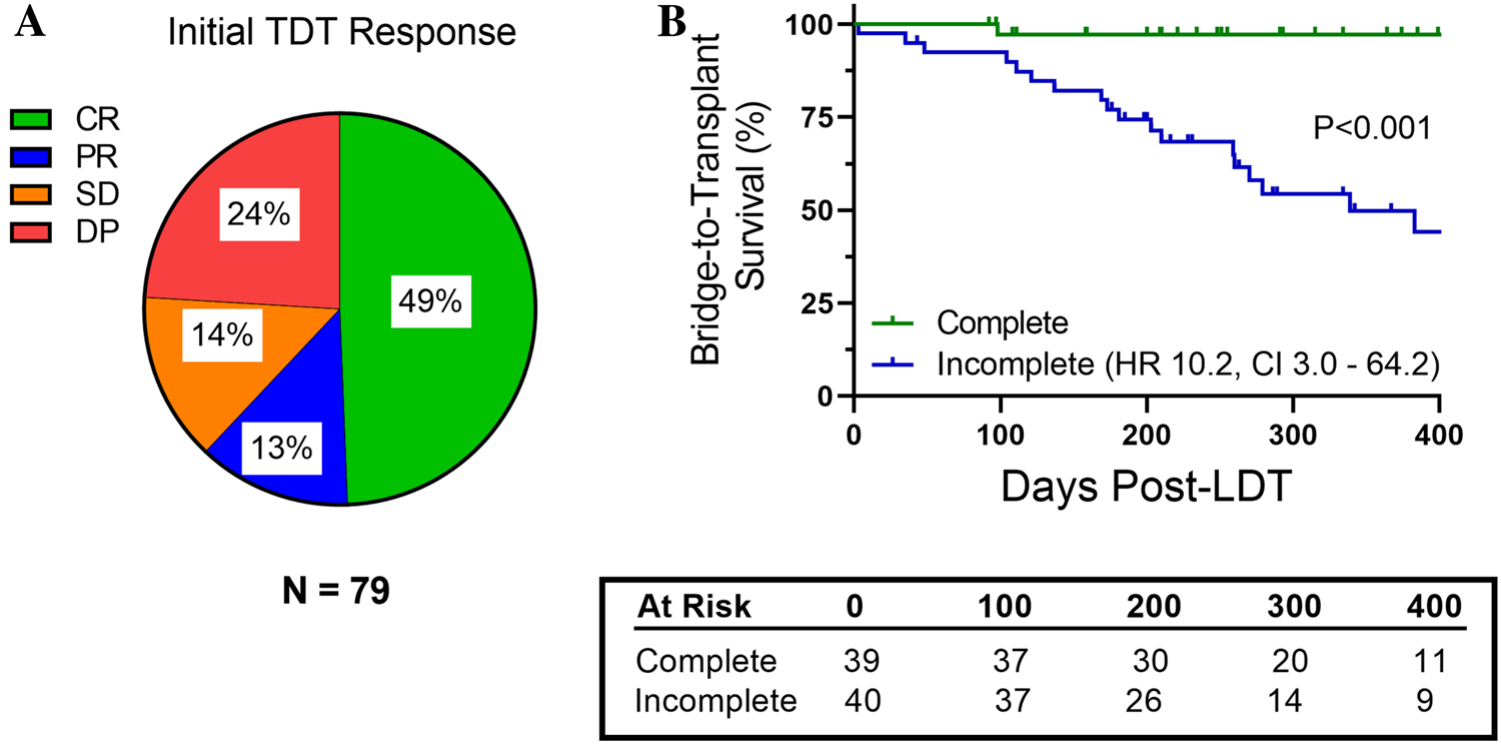
Response to initial liver-directed therapy and bridge-to-transplant survival. **A** mRECIST classification of cohort after initial liver-directed therapy: complete response (CR), partial response (PR), stable disease (SD), or disease progression (DP). **B** Kaplan–Meier curve of bridge-to-transplant survival based on response to initial liver-directed therapy as complete (CR) or incomplete (PR, SD, and DP). Hazard ratio (HR) and 95% confidence intervals (CI) are listed. Number of at-risk patients at each time interval is shown in table. Log-rank test was used to determine significance (*P* < 0.05)

We investigated incomplete response to initial LDT as a driving factor for bridge-to-transplant (BTT) survival due to known associations with increased risk for recurrence and higher tumor grade on explant pathology. Routine follow-up imaging prior to additional LDT was available for 79/86 patients in the cohort. Median follow-up imaging period for first-line LDT was as follows: DEB-TACE—62 days (IQR: 33–126 days), MWA—73 days (IQR: 38–129 days), and Y90—98 days (IQR: 90–170 days). Surveillance mRECIST without additional intervention at 3–6 months was available for 40/86 patients in the cohort, with 6/40 patients having a response class which differed from the initial mRECIST (Supplemental Table 1). Overall mRECIST classification is shown in Fig. 2A. Briefly, 49% of patients had a complete response (CR) in which the targeted lesion no longer displayed arterial enhancement after treatment, while 24% had disease progression (DP) indicated by > 30% arterial enhancement of targeted lesion or the development of new lesions meeting criteria for HCC.

Univariate analysis was conducted to evaluate the role of demographic factors as well as baseline clinical labs and radiographic measures of tumor burden in BTT outcomes following first-line LDT. Analysis revealed largest lesion size (OR 3.5, *P* < 0.001), being within Milan Criteria (OR 5.5, *P* = 0.028), AFP ≥ 50 ng/mL (OR 5.2, *P* = 0.012), and response to initial LDT [complete or incomplete] (OR 7.6, *P* = 0.003) were all significant risk factors impacting BTT outcome (Table 1). Multivariate analysis then showed AFP ≥ 50 ng/ml (OR 9.2, *P* = 0.018) and response to initial LDT (OR 7.4, *P* = 0.033) as risk factors for BTT outcomes.

Overall survival following LDT at 3-month, 6-month, and 1 year for cohort was 100%, 98%, and 80%. A total of 48 patients (56%) reached BTT endpoint. Kaplan–Meier survival analysis showed BTT survival for 3-month, 6-month, and 1-year at 97%, 86%, and 70%, respectively. Cox regression analysis of BTT survival showed HR 10.2, CI 3.0 – 64.2. Patients with a CR to initial LDT showed significantly superior BTT survival 3-month, 6-month, and 1-year BTT survival rates of 100%, 97%, and 97% compared to 95%, 77%, and 50% in those with incomplete responses (P < 0.001), (Fig. 2B).

### Clinical variables and response to initial liver-directed of therapy

A univariate analysis was then performed to identify pre-interventional variables that were associated with response to initial LDT (Table 2). Analysis showed general demographics, radiographic tumor burden (largest lesion size, cumulative lesion size, and multifocal burden), and measures of liver synthetic function (albumin, bilirubin, INR, and MELD-Na) were not associated with LDT response. As expected, AFP ≥ 50 ng/mL prior to LDT was associated with tumor response (*P* < 0.001). Complete blood counts revealed only absolute lymphocyte count (ALC) were associated with response to LDT (*P* = 0.009); patients with incomplete responses had significantly lower ALC (median 1.6 10^3^/mL, IQR: 1.0–1.9) prior to treatment compared to those with CR (ALC median 1.8 10^3^/mL, IQR: 1.4–2.7).

**Table 2:** Pre-Treatment Clinical Variables Associated with Incomplete Response to Initial LDT

			Univariate		Multivariate	
Demographics	Complete	Incomplete	*P* value	OR (95% CI)	*P* value	OR (95% CI)
Race (%)			0.948			
Caucasian	64	68				
African-American	31	27				
Other	5	5				
Age, years (IQR)	53 (57**–**68)	62.5 (59**–**66)	0.589			
Sex, %			0.869			
Male	69	68				
Female	31	32				
Cirrhosis etiology, %			0.675			
HCV	46	53				
HCV + EtOH	18	25				
NASH	13	10				
EtOH	10	7				
Other	13	5				
Tumor burden						
Multifocal, %	18	25	0.445			
Largest lesion size, cm (IQR)	2.7 (2.3**–**3.5)	3.3 (2.5**–**4.1)	0.228			
Cumulative lesion size, cm (IQR)	2.9 (2.4**–**4.3)	3.6 (2.7**–**5.0)	0.404			
Milan criteria, within, %	85	83	0.800	0.86 (0.26**–**2.8)		
Hepatocellular carcinoma biomarker						
AFP, ng/mL (IQR)	9.4 (5.8**–**29)	44.5 (7.5**–**190)	0.416			
AFP, ≥ 50 ng/mL			** < 0.001**	**6.6 (2.1–20.5)**	** < 0.001**	6.8 (2.1**–**22.0)
Laboratory values						
Sodium, mmol/L (IQR)	139 (137**–**141)	138 (136**–**140)	0.483			
Creatinine, mg/dL (IQR)	0.9 (0.8**–**1.1)	0.8 (0.8**–**1.1)	0.482			
Albumin, g/dL (IQR)	3.4 (2.9**–**3.7)	3.3 (2.8**–**3.6)	0.479			
Bilirubin, mg/dL (IQR)	0.9 (0.5**–**1.5)	1.1 (0.7**–**1.6)	0.679			
INR, (IQR)	1.1 (1.0**–**1.5)	1.1 (1.0**–**1.2)	0.521			
MELD-Na, at time of LDT (IQR)	10 (6**–**12)	11 (9**–**14)	0.646	1.0 (0.92**–**1.2)		
Immune laboratory values						
WBC, 10^3^/mL (IQR)	5.5 (4.2**–**7.3)	4.9 (4.0**–**7.0)	0.261			
PMN, 10^3^/mL (IQR)	3.0 (1.8**–**4.1)	2.7 (1.9**–**4.3)	0.782			
ALC, 10^3^/mL (IQR)	1.8 (1.4**–**2.7)	1.6 (1.0**–**1.9)	**0.009**	**0.44 (0.24–0.85)**	**0.017**	**0.44 (0.22–0.91)**
Monocytes, 10^3^/mL (IQR)	0.6 (0.5**–**0.7)	0.5 (0.3**–**0.7)	0.144			
Platelets, 10^3^/mL (IQR)	125 (95**–**198)	119.5 (87**–**170)	0.202			

Values are reported as percentage of total (%) or median (IQR). WBC, white blood cell counts; PMN, polymorphonuclear leukocytes; ALC, absolute lymphocytes. Variables from univariate with *P* < 0.05 were used for multivariate analysis

Bold value indicates *P* < 0.05

### T cell lineage and activation status in response to initial liver-directed therapy

**Fig. 3:**
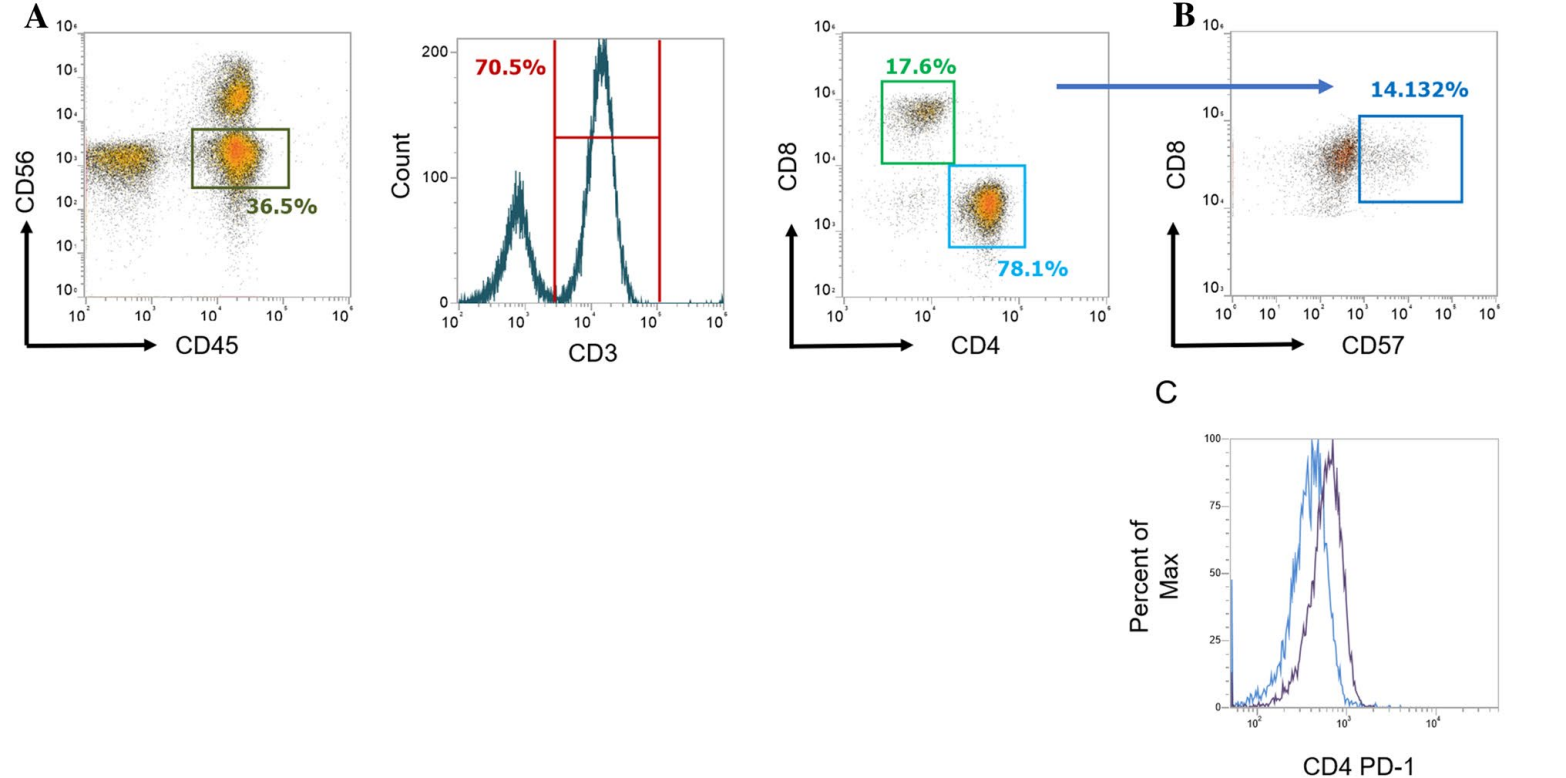
Gating strategy for *T* cell lineage and expression markers via flow cytometry. **A** PBMCs were stained then gated based on CD56^Negative^. Cells were then selected for CD3^Positive^ and plotted by CD4 and CD8 expression. Expression marker levels of CD57 and PD-1 were determined for CD4 and CD8. **B** Percentage of CD57 expression on CD4 and CD8 was determined. **C** Representative of median fluorescence intensity overlay of PD-1^Intermediate^ (purple) and PD-1^Low^ (blue) patient

Of the common clinical laboratory variables monitored for patients with HCC, ALC was found to be associated with initial response to LDT. Whether or not certain senescent or exhausted *T* cell phenotypes play a role in treatment-resistance was investigated. Expression of CD57 and PD-1 was determined using the gating strategies outline in Fig. 3. Neither CD4 nor CD8 *T* cells counts were associated with initial response (Table 3). CD57, a marker of *T* cell functional senescence, was also not associated with how the targeted lesion responded to initial LDT. However, univariate analysis revealed intermediate expression of PD-1 on CD4 and CD8 *T* cells was associated with initial response to LDT (*P* = 0.034 and *P* = 0.043, respectively, Table 3). Next, we performed a multivariate nominal logistic fit model with parameters significantly associated with response to initial LDT accounting to the univariate analysis (Table 3). In a multivariate logistic regression controlling for AFP ≥ 50 ng/ml, ALC (OR 0.46, *P* = 0.034) and intermediate expression of PD-1 on CD8 (OR 4.2, *P* = 0.018) remained predictive of response to initial LDT.

**Table 3:** Pre-Treatment analysis of T cell populations and incomplete response to initial LDT

			Univariate		Multivariate	
Variables	Complete	Incomplete	*P* value	OR (95% CI)	*P* value	OR (95% CI)
AFP, ≥ 50 ng/mL			** < 0.001**	**6.6 (2.1–20.5)**	** < 0.001**	**9.0 (2.6 – 31.3)**
ALC, 10^3^/mL (IQR)	1.8 (1.4**–**2.7)	1.6 (1.0**–**1.9)	**0.009**	**0.44 (0.24–0.85)**	**0.034**	**0.46 (0.21 – 0.98)**
T cell Phenotypes						
CD4, % of CD3 (IQR)	76.8 (68.9**–**92.2)	77.2 (70.2**–**82.7)	0.996			
CD4, 10^3^/mL (IQR)	0.9 (0.6**–**1.3)	0.74 (0.44**–**1.1)	0.076			
CD8, % of CD3 (IQR)	15.6 (11.8**–**24.1)	15.2 (11.1**–**22.9)	0.983			
CD8, 10^3^/mL (IQR)	0.18 (0.12**–**0.34)	0.16 (0.08**–**0.21)	0.131			
CD4:CD8, ratio (IQR)	5.0 (2.9**–**6.9)	5.5 (3.3**–**7.6)	0.435			
CD57 + CD4 + , % of CD4 (IQR)	2.7 (1.5**–**7.2)	2.9 (1.9**–**6.9)	0.936			
CD57 + CD8 + , % of CD8 (IQR)	24.6 (17.8**–**33.2)	25.6 (13.3**–**41.6)	0.691			
PD-1 + CD4 + , ≥ 460 MFI			**0.034**	**3.3 (1.03–10.3)**		
PD-1 + CD8 + , ≥ 600 MFI			**0.043**	**3.0 (0.99–8.8)**	**0.018**	**4.2 (1.2–14.8)**

Values ALC, absolute lymphocyte count; PD-1, programmed cell death protein-1. Variables from univariate with *P* < 0.05 were used for multivariate analysis

Bold value indicates *P* < 0.05

### Decompensation of cirrhosis history and decreases in lymphocyte levels independent of active viral hepatitis

We next analyzed the history of the cohort prior to HCC diagnosis to examine changes in the ALC across cirrhotic disease progression and the diagnosis of HCC prior to LDT. ALC values were extracted from the electronic medical record at three different time points for each patient: earliest available in record (median 1871 days prior to LDT), day of HCC diagnosis (median 86 days prior to LDT), and day of initial LDT. Analysis showed a significant difference in ALC with a downward trend upon HCC diagnosis (*P* = 0.029, Supplemental Fig. 1), however post hoc analysis revealed no differences between the mean of each ALC timepoint. These results indicate ALC is stable prior to HCC development.

To further investigate cirrhosis-associated immune dysfunction, we investigated whether decompensating events were associated with ALC or PD-1 expression on *T* cells. Patients that experienced a decompensating event had significantly lower lymphocyte counts (*P* < 0.001) while no associations were found for PD-1 expression on either CD4 or CD8 *T* cells (Supplemental Table 3, *P* = 0.225 and *P* = 0.273). Patients with cirrhosis complications that included either ascites, hepatic encephalopathy, or bleeding esophageal varices were associated with significantly lower ALC prior to LDT (Supplemental Table 4, *P* < 0.001, *P* < 0.001, and *P* = 0.03, respectively). Patients diagnosed with portal hypertension and splenomegaly also had significantly lower ALC (*P* = 0.002 and *P* < 0.001, respectively). Additionally, a weak association was observed between MELD-Na and ALC prior to LDT (*P* = 0.003, *R*^2^ = 0.10). While active viral hepatitis infections can impact *T* cell counts, no associations between viral hepatitis status at the time of LDT (active, sustained virologic response, or non-viral) and ALC or PD-1 expression was found (Supplemental Table 3).

### Liver-directed therapy-induced fluctuations in PD-1 expression

**Fig. 4:**
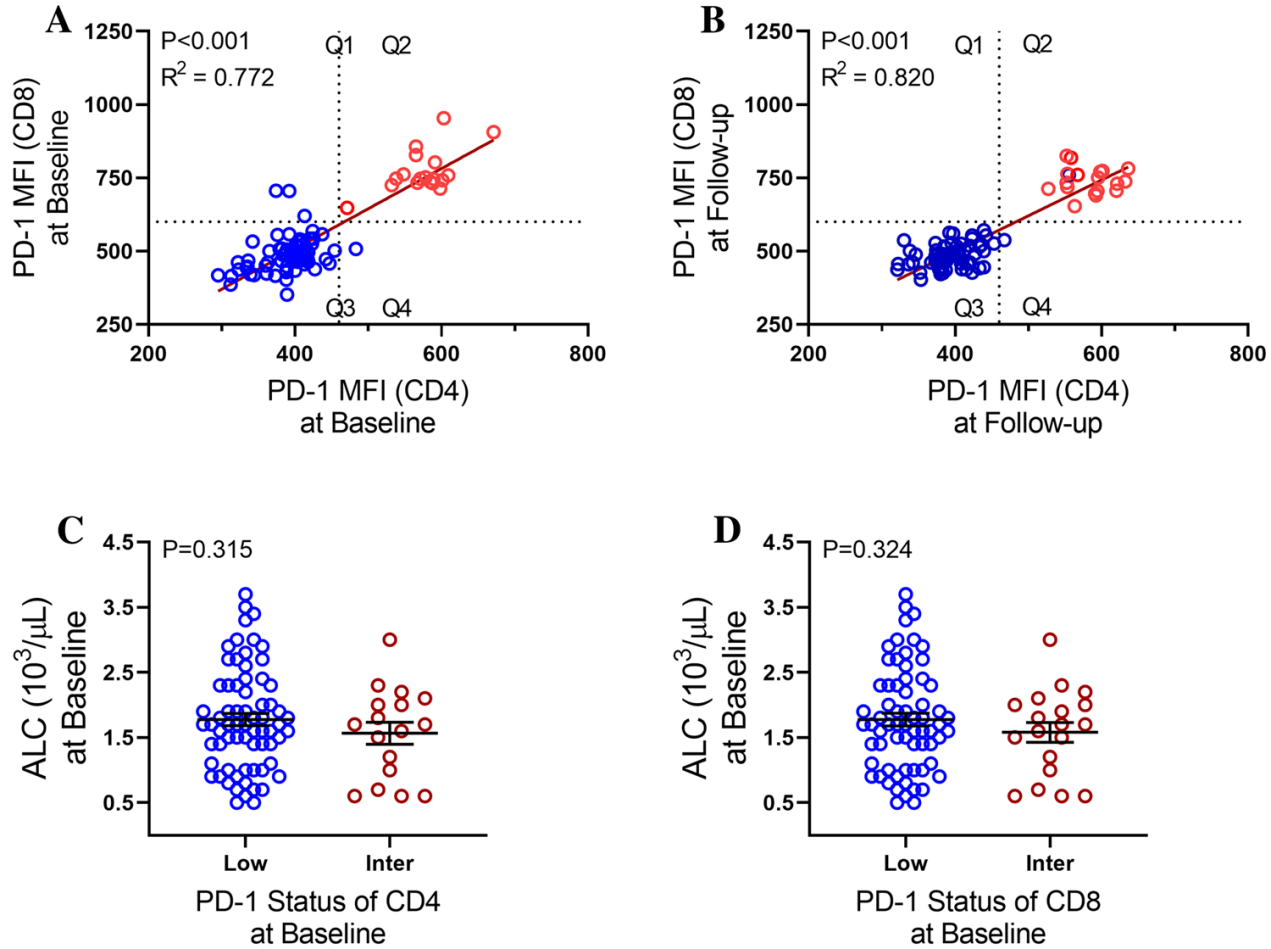
PD-1 expression on CD4 and CD8 before and after liver-directed therapy and relationship with ALC prior to intervention. Correlation between PD-1 expression on CD4 and CD8 at **A** baseline and **B** follow-up based on median fluorescence intensity (MFI). Two populations of PD-1 expression emerged, PD-1^Intermediate^ (Q2) and PD-1^Low^ (Q3). ALC at baseline with **C** PD-1 CD4 status and **D** PD-1 CD8 status. Data represent mean ± SEM

Successful treatment may reduce *T* cell exhaustion and increase anti-tumoral response. We investigated PD-1 expression on both CD4 and CD8 *T* cells prior to and following LDT. A strong positive correlation was found between expression of PD-1 on CD4 and CD8 *T* cells that persisted post-LDT regardless of response to treatment (Fig. 4A and B). Two distinct populations were visible (Q2 and Q3) and grouped as low (Q3) and intermediate (Q2) PD-1 expression (Fig. 4A and B). Cutoffs for PD-1^Intermediate^ expression were determined for both CD4 (460 MFI) and CD8 (600 MFI). All patients with intermediate PD-1 expression on CD8 also had intermediate PD-1 expression on CD4. No association was found between ALC and PD-1 status on CD4 (Fig. 4C) or CD8 (Fig. 4D) prior to LDT.

**Fig. 5:**
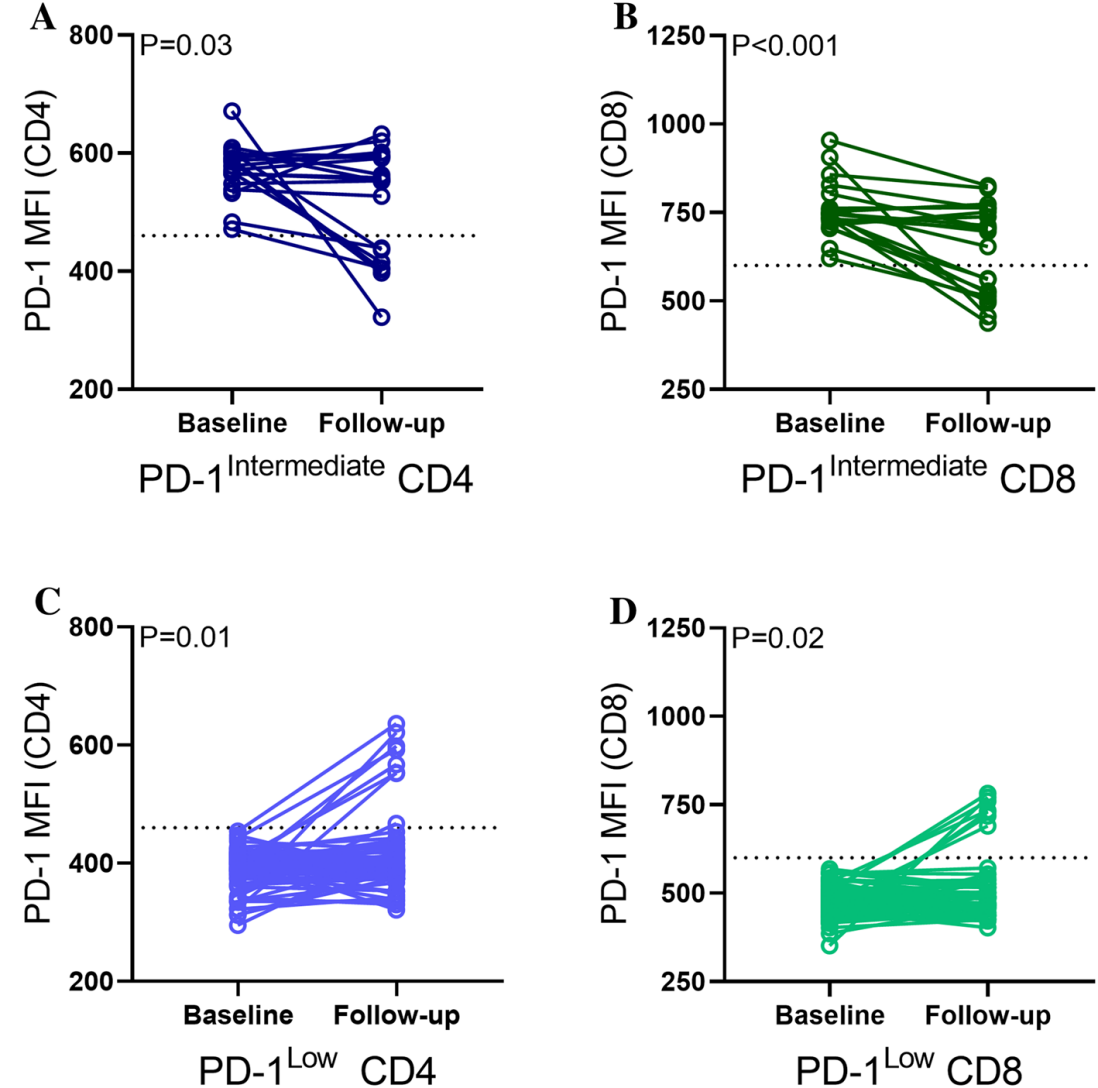
Liver-directed therapy induced changes in PD-1 expression on CD4 and CD8. **A** PD-1^Intermediate^ CD4 and **B** PD-1^Intermediate^ CD8 expression at baseline and following LDT. **C** PD-1^Low^ CD4 and **D** PD-1^Low^ CD8 expression at baseline and following LDT. A matched paired *t* test was used to determine significance

Matched paired analysis did not reveal a treatment effect on PD-1 levels (intermediate or low on either CD4 or CD8 *T* cells) regardless of treatment modality. We next sought to determine whether PD-1 expression changed between intermediate and low expressers. Regardless of treatment modality, LDT caused a decrease in PD-1 expression for patients with intermediate PD-1 at baseline for CD4 (*P* = 0.03) and CD8 (*P* < 0.001) (Fig. 5A and B). Patients with low PD-1 expression on CD4 or CD8 did change in expression after LDT (*P* = 0.01 and *P* = 0.02, respectively) (Fig. 5C and D). Several patients switched PD-1 expression class from low to intermediate or vice versa following treatment.

### Intermediate PD-1 expression and bridge-to-transplant survival

**Fig. 6:**
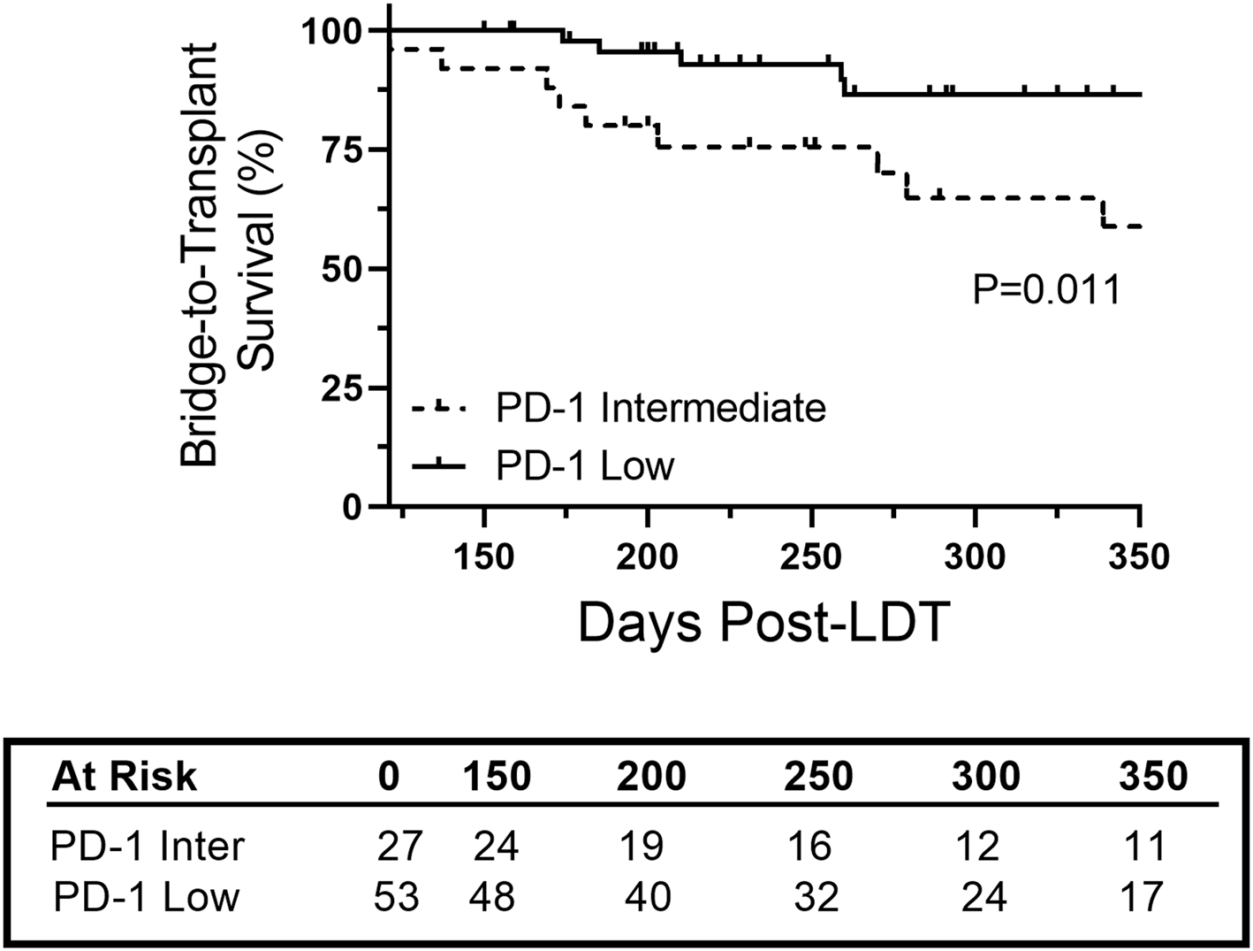
PD-1 expression status and bridge-to-transplant survival. Kaplan–Meier curve of bridge-to-transplant survival based on PD-1 expression status during initial liver-directed therapy. Number of at-risk patients at each time interval is shown in table. Log-rank test was used to determine significance

Based on alterations in PD-1 expression post-treatment, patients were classified as PD-1^Intermediate^ if either baseline or follow-up sample was above thresholds of 460 (CD4) and 600 (CD8) MFI. Bridge-to-transplant survival was then investigated after 120 days to account for changes in PD-1 expression following LDT. The overall 3-month, 6-month, and 1-year survival was 100%, 91%, and 74%, respectively. Survival for PD-1^Intermediate^ group at 3-month, 6-month, and 1-year survival was 100%, 84%, and 59% (*P* = 0.011) compared to 100%, 98%, and 87% in PD-1 low group (Fig. 6).

### PD-1^Intermediate^ expression and explant pathological tumor grade

In the cohort, 48 patients reached endpoint of which 24 (50%) were transplanted. Pathological grading (TNM staging) classification was available for 18/24 (75%) patients with remaining six having indeterminant staging due to complete tumor necrosis. Viable lesions and presence of either lymphovascular invasion or satellite nodules was associated with pathological TNM staging. Patients with intermediate PD-1 expression on both CD4 and CD8, 2/4 patients had higher TNM staging on explant (*P* < 0.005) compared to those with PD-1 low 0/15 (0%) (Supplemental Table 5).

## Discussion

The benefits of tumor response to LDT in bridge-to-transplant HCC has been well documented, however, baseline factors associated with optimal first-line response to LDT are limited to radiographic burden. Our results demonstrate that patients with a complete response to initial LDT, regardless of treatment modality, had superior BTT survival and that baseline circulating lymphocyte count and *T* cell PD-1 expression are critical factors influencing response to first-line LDT.

The overall objective response (CR or PR) rate to first-line LDT in this study was 72%, which is in agreement with other recent studies of LDT response rates 72–88% [6, 18]. A complete radiographic response was seen in 49% of these patients. Over half of the cohort (64%, 55/86) required additional LDT prior to BTT endpoint. Complete responses to LDT are often not sustained after a single treatment. Within patients with a CR, 38% (15/39) required additional LDT of the primary or in a new lesion. A study that investigated sustained CR rates after DEB-TACE found only 62% remained with complete radiographic response after 6 months and only 31% after one year [19]. Despite this, complete radiographic response to first-line LDT was associated with extended bridge-to-transplant survival highlighting the relationship between initial response and extended time to tumor progression (Complete Response: 994 days vs Incomplete Response: 339 days). Although lesion size has been known to be predictive of response to LDT, our results, through multivariate analysis, show response to initial LDT was more predictive of BTT survival than lesion size alone, with an adjusted HR of 10.2. Given the superior post-LDT outcomes in patients with an initial complete response, identifying patient-specific factors related to HCC biology and progression of cirrhosis associated with initial complete response are paramount.

In this prospective cohort study, we focused on baseline clinical labs and MELD-Na score prior to LDT which revealed lymphocyte count as a critical variable in the initial response to LDT. Patients with initial CR had significantly higher *T* cell counts than those with incomplete responses, suggesting *T* cell-mediated anti-tumor immunity could play a significant role in the response to LDT. Radiographic measures of tumor burden were not associated with initial response, further suggesting that tumor biology, cirrhotic immune status, and possibly the target action of the LDT modality may be more critical determinants of response in early stage HCC. A recent retrospective study of 93 consecutive HCC patients treated with DEB-TACE and underwent liver transplant, found high ALC values were associated with favorable (CR or PR) responses [4]. Indices that incorporate lymphocyte counts such as neutrophil-to-lymphocyte ratio and platelet-to-lymphocyte ratio may be valuable tools for prognosis of tumor response [20, 21] and OS following LDT [22, 23], but it is unclear whether the strength of these indices may be driven solely by the lymphocyte count. Our results did not find any associations between neutrophils or platelets with response to LDT demonstrating lymphocyte count may be the driving force in the indices.

Liver-directed therapies generate necrosis and the release of tumor neoantigens which can elicit an anti-tumoral immune response [24–26]. However, chronic antigen stimulation (viral hepatitis) and immune exhaustion (cirrhosis-associated immune dysfunction) render the neoantigen response ineffective. PD-1 expression is elevated following *T* cell activation through *T* cell receptor-dependent and independent mechanisms (see review [27]). Ligation of PD-1 and other co-inhibitory receptors inhibits *T* cell activation by altering cytokine production, proliferation, and cellular metabolism. Persistent antigen stimulation, as in the case with chronic inflammation and cancer, can cause *T* cell exhaustion and high levels of PD-1 expression (see reviews [27, 28]). Once the antigen is cleared, PD-1 expression returns to basal levels (see review [29]). Most of the cohort, 71% (61/86), had HCV-related HCC of which 61% (37/61) had active HCV infection at the time of initial LDT. While chronic HCV infections are known to cause *T* cell dysfunction (see review [30]), PD-1 expression and lymphocyte counts were not associated with viral hepatitis status. As expected, patients with decompensated cirrhosis had significantly lower ALC compared to those patients without decompensation. Cirrhosis-associated immune dysfunction arises during the progression from compensated to decompensated and can lead to a relative immunodeficiency state (as reviewed by [31]). Patients with portal hypertension with splenomegaly had significantly lower peripheral lymphocyte counts, which may impact response to LDT independent of HCV infection.

Liver-directed therapy impacts the immune system by not only releasing tumor-associated antigens (TAA) but can cause lymphopenia. The effect of LDT-induced changes in circulating *T* cells expressing PD-1 remains unknown. In our cohort, the effect of LDT on PD-1 expression was not universal for all patients. In our patients with elevated PD-1, some experienced a decrease in PD-1 expression while some patients remained or became elevated after treatment. Elevated PD-1 expression was associated with incomplete responses to LDT and shorter bridge-to-transplant survival. These patients may be more responsive to treatment with the addition of PD-1 blockade. A study investigating PD-1 expression in tumor-infiltrating *T* cells found those with high PD-1 expression were also enriched in *T* cell exhaustion markers [32] potentially limiting the effect of anti-tumoral responses generated by LDT. Currently, two anti-PD-1 immunotherapies, pembrolizumab, and nivolumab, are FDA approved for second-line treatment of advanced stage HCC. In a small cohort of 21 patients with advanced stage HCC, responders to nivolumab or pembrolizumab had elevated PD-1 expression on intratumoral CD8 *T* cells while those with tumor progression had elevated expression on circulating *T* cells [33]. The effectiveness of anti-PD-1 therapy was shown in a study with tumor-infiltrating CD8 with high PD-1 expression treated with anti-PD-1 and anti-TIM3 and/or LAG3 therapy had restored hindered proliferation and cytokine production [32]. Combination therapy of LDT with immunotherapy is intriguing as PD-1 blockade could render *T* cells more responsive to neoantigens generated by LDT. A number of clinical trials evaluating the effect of anti-PD-1 immunotherapy in combination with LDT are underway as phase 1 trials for intermediate-advanced HCC not amenable for resection or OLT (NCT02837029, NCT03143270, and NCT03099564) with several phase two trials completed late 2019 (NCT03380130, NCT03033446) with results pending.

Biomarkers to predict anti-PD-1 immunotherapy response for HCC are limited. Several studies have investigated the use of PD-L1 expression on HCC tumors to predict response to anti-PD-1 therapy. However, expression of PD-L1 on HCC tumors was found to have both inter-assay heterogeneity and spatial variance within the tumor [34]. In the clinical trial Checkmate-040 for nivolumab, response rate did not correlate with PD-L1 expression on tumors further demonstrating the issue that PD-L1 expression on the tumor does not equate to favorable anti-PD-1 response rates. Also, it is not feasible to obtain biopsies on all HCC tumors to determine PD-L1 expression as this increases the risk of tumor seeding. Our results determine PD-1 expression on circulating *T* cells following HCC diagnosis prior to therapy and provides immediate insight into which patients would benefit from anti-PD-1 immunotherapy.

Limitations of the study include the single-center nature at a short-wait time (< 120 days) transplant center. However, a benefit for this single-center study is the consistent institutional protocols and experience with LDT. Although this study investigated multiple LDT modalities, treatment approach may play a significant role in the post-treatment immune landscape and require a larger cohort powered to assess the role of first-line LDT which is the subject of future research. While changes in PD-1 expression could be observed at primary treatment follow-up (~ 30 days), changes from baseline may be magnified by complete HCC necrosis, HCC progression, or progression of cirrhosis. The changes may also be magnified at centers with a longer wait time and could have greater implications on bridge-to-transplant outcomes warranting further study.

The mechanisms that limit the effectiveness of first-line LDT remain poorly understood, but greatly impacts outcomes for HCC. Lymphocyte counts, which are monitored throughout waitlist time for OLT, may have an increased role in predicting both patient’s response to treatment and BTT survival. Patients presenting with low lymphocytes and elevated PD-1 expression may benefit from a combination of immunotherapy and LDT to boost anti-tumoral immune response in hopes of improving overall post-transplant survival.

### Supplementary Information

Below is the link to the electronic supplementary material.

Supplementary file1 (DOCX 49 KB)

## Data Availability

The datasets generated and analyzed in the study are available upon request from the corresponding author PT.
