# Supplementary material for: PD-1 expression in hepatocellular carcinoma predicts liver-directed therapy response and bridge-to-transplant survival
Source: Cancer Immunol Immunother. 2021 Oct 24;71(6):1453–65. doi: 10.1007/s00262-021-03087-z (PMC9122885; doi:10.1007/s00262-021-03087-z)
Supplement: Supplementary file 1 — Supplementary file1 (DOCX 49 KB) [file 262_2021_3087_MOESM1_ESM.docx]

SUPPLEMENTAL TABLES

**Supplemental Table 1** Cohort demographics, baseline clinical variables, and response to liver-directed therapy

|  | Cohort, n=86 |
| --- | --- |
| **Study timeframe** | August 2016 - March 2020 |
| **Demographics** |  |
| **Race, n (%)** |  |
| Caucasian | 56 (65) |
| African American | 24 (28) |
| Other | 6 (7) |
| **Age, years (IQR)** | 63 (58 - 66) |
| **Sex, n (%)** |  |
| Male | 60 (70) |
| Female | 26 (30) |
| **Cirrhosis etiology, n (%)** |  |
| HCV | 44 (51) |
| HCV+EtOH | 17 (20) |
| NASH | 11 (13) |
| EtOH | 7 (8) |
| Other | 7 (8) |
| **Tumor burden** |  |
| **Multifocal, n (%)** | 20 (23) |
| **Largest lesion size, cm (IQR)** | 3.0 (2.4 - 3.8) |
| **Cumulative lesion size, cm (IQR)** | 3.5 (2.6 - 5.0) |
| **Milan criteria, within, n (%)** | 70 (81) |
| **Hepatocellular carcinoma biomarker** |  |
| **AFP, ng/mL (IQR)** | 15.5 (6.2 - 114) |
| **AFP ≥50 ng/mL n (%)** | 28 (34) |
| **Laboratory values** |  |
| **Sodium, mmol/L (IQR)** | 139 (137 - 141) |
| **Creatinine, mg/dL (IQR)** | 0.8 (0.8 - 1.1) |
| **Albumin, g/dL (IQR)** | 3.4 (2.9 - 3.6) |
| **Bilirubin, mg/dL (IQR)** | 1.0 (0.6 - 1.5) |
| **INR, (IQR)** | 1.1 (1.0 - 1.2) |
| **MELD-Na, at time of LDT (IQR)** | 10 (8 - 12) |
| **Liver-directed therapy** |  |
| **Initial liver-directed therapy, n (%)** |  |
| DEB-TACE | 38 (44) |
| Y90 | 27 (31) |
| MWA | 21 (25) |
| **mRECIST to initial LDT, n (%)** |  |
| Complete | 39 (49) |
| Partial | 10 (13) |
| Stable | 11 (14) |
| Progression | 19 (24) |
| **Bridge-to-Transplant endpoint** |  |
| **Liver transplant, n (%)** | 24 (28%) |
| **Tumor progression, n (%)** | 24 (28%) |

Values are reported as n (%) or median (IQR). HCV, hepatitis C virus; EtOH, alcoholic steatohepatitis, NASH, nonalcoholic steatohepatitis; AFP, alpha-fetoprotein; INR, international normalized ratio; MELD-Na, model of end stage liver disease sodium; DEB-TACE, doxorubicin-eluting bead transarterial chemoembolization; Y90, Yttrium-90; MWA, microwave ablation; mRECIST, modified response evaluation criteria in solid tumors; LDT, liver-directed therapy

| **Supplemental Table 2**. Initial vs durable mRECIST scores from first-line tumor-directed therapy | | | | | |
| --- | --- | --- | --- | --- | --- |
|  |  | Durable mRECIST | | | |
|  |  | CR | PR | SD | DP |
| Initial mRECIST | CR | 28 | 0 | 0 | 3 |
|  | PR | 0 | 1 | 1 | 0 |
|  | SD | 0 | 0 | 1 | 1 |
|  | DP | 0 | 0 | 1 | 4 |

**Supplemental Table 3** Decompensating Events and Viral Hepatitis Status Associates with ALC

|  | **ALC, 10^3^/mL** | | **PD-1 CD4, MFI** | | **PD-1 CD8, MFI** | |
| --- | --- | --- | --- | --- | --- | --- |
|  | **Median (IQR)** | **P-value** | **Median (IQR)** | **P-value** | **Median (IQR)** | **P-value** |
| **Decompensating event** |  | **<0.001** |  | 0.225 |  | 0.273 |
| Absent | 1.9 (1.5 - 2.6) |  | 406 (391 - 488) |  | 509 (464 - 712) |  |
| Present | 1.5 (0.5 - 1.8) |  | 402 (375 - 425) |  | 500 (457 - 557) |  |
| **Viral hepatitis status** |  | 0.067 |  | 0.545 |  | 0.866 |
| Active | 1.8 (1.6 - 2.4) |  | 400 (375 - 448) |  | 500 (442 - 564) |  |
| Sustained virologic response | 1.6 (1.1 - 2.3) |  | 410 (379 - 423) |  | 507 (468 - 620) |  |
| Non-viral | 1.5 (0.9 - 1.7) |  | 405 (389 - 540) |  | 496 (461 - 717) |  |

**Supplemental Table 4** Cirrhosis-Associated Complications and ALC

|  | **ALC (10^3^/mL)** | |
| --- | --- | --- |
| **Cirrhosis Complications** | **Median (IQR)** | **P-value** |
| **Ascites** |  | **<0.001** |
| Yes | 1.2 (0.9 - 1.7) |  |
| No | 1.9 (1.5 - 2.4) |  |
| **Hepatic encephalopathy** |  | **<0.001** |
| Yes | 1.2 (0.8 - 1.7) |  |
| No | 1.8 (1.5 - 2.4) |  |
| **Esophageal varices (w/bleeding)** |  | **0.030** |
| Yes | 1.4 (0.8 - 1.8) |  |
| No | 1.7 (1.5 - 2.3) |  |
| **Jaundice** |  | 0.276 |
| Yes | 1.6 (0.8 - 2.2) |  |
| No | 1.7 (1.1 - 2.3) |  |
| **Portal hypertension** |  | **0.002** |
| Yes | 1.6 (0.9 - 1.9) |  |
| No | 1.9 (1.5 - 2.7) |  |
| **Splenomegaly** |  | **<0.001** |
| Yes | 1.3 (0.9 - 1.6) |  |
| No | 2.0 (1.7 - 2.7) |  |

**Supplemental Table 5** PD-1 High Expression with Pathological TNM Staging

| **Variables** | **No. transplanted** | **T1-T2** | **T3-T4** | **P-value** |
| --- | --- | --- | --- | --- |
| **Viable lesions at explant, n (%)** | 18 | 14 (93) | 3 (100) | 0.753 |
| **Presence of lymphovascular invasion, n (%)** | 18 | 1 (7) | 0 (0) | 0.540 |
| **Presence of satellite nodules, n (%)** | 18 | 2 (13) | 1 (33) | 0.431 |
| **PD-1 CD4 and CD8 High, n (%)** | 6 | 3 (50) | 3 (50) | **0.005** |
| **PD-1 CD4 and CD8 Inter/low, n (%)** | 12 | 12 (100) | 0 (0) |  |

Values are reported as n, number of total and %, percentage of total

**Supplemental Fig 1** Absolute lymphocyte count prior to tumor-directed therapy. ALC was extracted from electronic medical record at three timepoints, earliest within records, at HCC diagnosis, and day of TDT. Error bars are shown as median with interquartile range. One-way ANOVA showed significant difference in ALC (P=0.029) however, post hocs analysis revealed no significant difference.
